# Metabolites of pathogenic microorganisms database (MPMdb) and its seed metabolite applications

**DOI:** 10.1128/spectrum.02342-23

**Published:** 2024-02-23

**Authors:** Feng Jiang, Yao Ruan, Xiao-Hui Chen, Hai-Long Yu, Ting Cheng, Xin-Ya Duan, Yan-Guang Liu, Hong-Yu Zhang, Qing-Ye Zhang

**Affiliations:** 1 Hubei Key Laboratory of Agricultural Bioinformatics, College of Informatics, Huazhong Agricultural University, Wuhan, China; Instituto de Ecología, A.C. (INECOL), Pátzcuaro, Mexico

**Keywords:** pathogenic microorganisms, metabolites, seed metabolites, evolution taxonomy, auxotroph analysis, specific bactericide designing

## Abstract

Seed metabolites are the combination of essential compounds required by an organism across various potential environmental conditions. The seed metabolites screening framework based on the network topology approach can capture important biological information of species. This study aims to identify comprehensively the relationship between seed metabolites and pathogenic bacteria. A large-scale data set was compiled, describing the seed metabolite sets and metabolite sets of 124,192 pathogenic strains from 34 genera, by constructing genome-scale metabolic models. The enrichment analysis method was used to screen the specific seed metabolites of each species/genus of pathogenic bacteria. The metabolites of pathogenic microorganisms database (MPMdb) (http://qyzhanglab.hzau.edu.cn/MPMdb/) was established for browsing, searching, predicting, or downloading metabolites and seed metabolites of pathogenic microorganisms. Based on the MPMdb, taxonomic and phylogenetic analyses of pathogenic bacteria were performed according to the function of seed metabolites and metabolites. The results showed that the seed metabolites could be used as a feature for microorganism chemotaxonomy, and they could mirror the phylogeny of pathogenic bacteria. In addition, our screened specific seed metabolites of pathogenic bacteria can be used not only for further tapping the nutritional resources and identifying auxotrophies of pathogenic bacteria but also for designing targeted bactericidal compounds by combining with existing antimicrobial agents.

**IMPORTANCE:**

Metabolites serve as key communication links between pathogenic microorganisms and hosts, with seed metabolites being crucial for microbial growth, reproduction, external communication, and host infection. However, the large-scale screening of metabolites and the identification of seed metabolites have always been the main technical bottleneck due to the low throughput and costly analysis. Genome-scale metabolic models have become a recognized research paradigm to investigate the metabolic characteristics of species. The developed metabolites of pathogenic microorganisms database in this study is committed to systematically predicting and identifying the metabolites and seed metabolites of pathogenic microorganisms, which could provide a powerful resource platform for pathogenic bacteria research.

## INTRODUCTION

Pathogenic microorganisms seriously threaten food security, regional economy, human community structures, and the biodiversity of natural ecosystems worldwide (1–4). Meanwhile, the large-scale application of bactericides has led to the emergence of drug-resistant bacteria and even super bacteria (5). Comprehensive characterization and knowledge of pathogenic mechanisms of pathogenic microorganisms are critical for the effective prevention and control of infectious diseases (6).

A large number of pathogenic microorganisms live in complex and diverse environments where they compete for limited space and resources for survival (7). For example, faced with extreme cold, drought, and other extreme environments, *Pseudomonas syringae* will adopt the survival strategy by adjusting the metabolic profile to cope with extreme environments (8). Hence, the metabolism interactions between host and pathogen influence both bacterial virulence and host responses, thus driving the evolution, eventually resulting in the production of new pathogenic characteristics (9, 10). Many studies have demonstrated that metabolism is not only crucial for bacterial replication but also is closely related to maintaining the host’s infection state, completing bacteria’s life cycle, and overcoming the harsh living conditions caused by drug application (11, 12). Therefore, exploring the metabolic characteristics of pathogenic bacteria and their interaction with the environment is essential for understanding pathogenic bacteria and their pathogenic mechanism.

**Fig 1:**
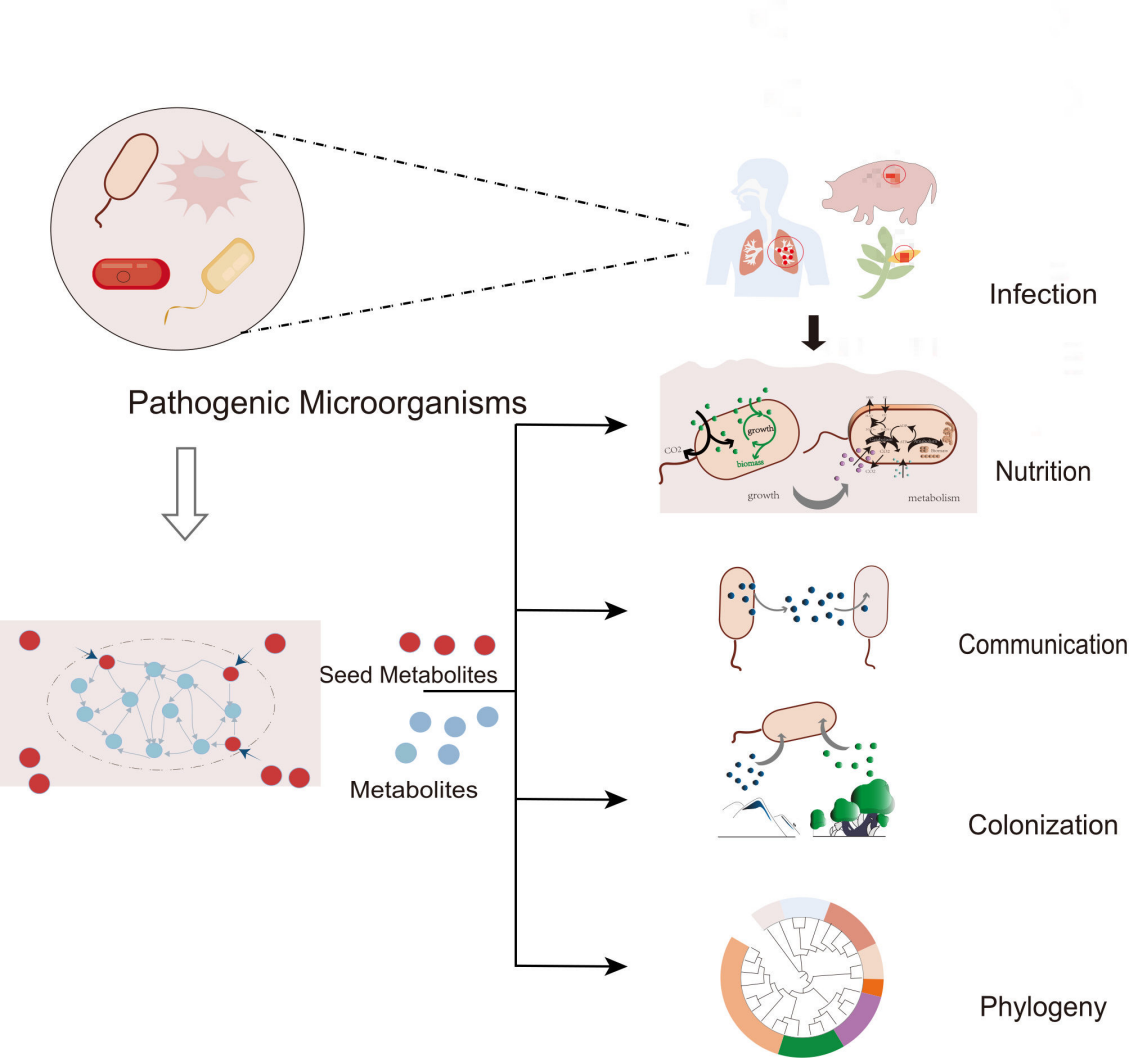
Wide applications of seed metabolites in pathogenic bacteria research.

The investigation into the metabolic traits of pathogenic bacteria and their interactions with environmental factors can be initiated by systematically identifying the seed metabolites (13–16) inherent to these organisms. The concept of seed metabolites refers to a crucial set of metabolites that are essential for the activation of all metabolic pathways in a microbial network, regardless of their dynamic activation under specific environmental conditions (Fig. 1). Seed metabolites encompass both essential metabolites, which lack biosynthetic pathways (also known as auxotrophies), and nutritive metabolites, which possess degradation pathways but lack synthesis routes in the genome. They represent the necessary compounds for activating the entire metabolic network across various potential environmental conditions, not just those actively engaged in a given context. Hence, seed metabolites constitute a comprehensive set of compounds vital for the survival and metabolic processes of microbes in diverse environmental settings. This set can be seen as the union of essential compounds needed across all these environments, reflecting the overall, static metabolic “interface” or “potential” of an organism, and serving as a proxy for its effective biochemical habitat.

Seed metabolites reveal the environmental factors affecting microbial colonization. As demonstrated by Borenstein et al. (17), a significant correlation exists between the diversity and size of seed metabolite sets in 478 species and environmental variations, indicating species in diverse environments possess larger and more varied seed metabolite sets (17). Seed metabolites are also pivotal in studying metabolic communication between species. Such communication often occurs within microbiomes and between organisms and hosts (18). Studying metabolic communication among microbial communities, especially gut microorganisms, reveals ecological relationships between microorganisms and the environment, offering new insights into microbial community stability and disease pathogenesis. Gonçalves et al. (19) utilized seed metabolites to investigate the metabolic interaction between *Ca liberbacter* and its host, finding that *Ca liberbacter* relies on the host (Citrus and woodlice) for essential nutrients and metabolites (19), a finding echoed by other studies. Understanding host-microorganism interactions aids in developing novel disease control strategies (19). Based on each species’ seed metabolites, Levy et al. (20) proposed two metabolic interaction indices, namely the metabolic competition index and the metabolic complementarity index, to predict interactions within microbiomes, elucidating microbial community assembly rules (20). This holds significant importance for understanding the basic ecological processes among microorganisms and the clinical treatment of diseases caused by microbial community imbalances.

However, a majority of microbial species remain uncultivated, and large-scale determination of pathogenic bacteria metabolomes is highly costly, which poses a large challenge to our understanding of the metabolic characteristics of pathogenic microorganisms (21). Rapid advancements in sequencing and bioinformatics technology have enabled the assembly and analysis of reference genomes for a wide range of pathogenic bacteria from diverse environments, which lays a foundation for organism’s metabolic reconstructions (22). In recent years, the genome-scale metabolic models (GSMMs) have been widely applied to study the metabolic characteristics of species.

GSMMs are a holistic representation of an organism’s metabolic network, mapping all metabolic reactions based on genomic data. It is used for predicting metabolic functions and responses to environmental changes, serving as a key tool in systems biology and biotechnological research (23). A frequently used strategy is to express the metabolic network as a directed graph for the analysis of the metabolic network. The topological structure of networks can directly reflect the network formation and evolution (24). Research showed topology analysis of metabolic networks focuses on network robustness or metabolic phenotype, uncovers potential pathophysiology mechanisms, predicts the viability of mutant strains, and determines cellular regulation. The genome-scale metabolic network can be used to identify seed metabolites (13–16). Each point (Fig. 1) in the graph represents the compound involved in the metabolic reaction, and its edge represents the reaction between the substrate and the product. Research suggests that seed metabolites based on metabolic networks can not only provide important information for the metabolic capacity and growth characteristics of species but also provide important insights for the evolution mechanism of species involved in metabolism as well as environmental changes (17, 25, 26).

In summary, the sets of seed metabolites not only depict the metabolic environment of a species but also offer deep insights into its inherent biochemical habitat and evolutionary traits (9). These characteristics represent a composite outcome of the organism’s encounters with various metabolic environments. The current research on seed metabolites mainly focuses on the characteristics of a single pathogen or the communication among microorganisms. So far, no study has been established to comprehensively collect and analyze the seed metabolites of all pathogenic bacteria. In addition, the huge cost of batch determination and metabolomics technological bottleneck limits the identification of metabolites, thus hindering the specific research on the type and distribution of seed metabolites of pathogenic bacteria.

In this study, we first explore the biological importance of seed metabolites of common pathogenic microorganisms obtained from a network topology-based approach. To comprehensively identify the correlation between the fraction of seed metabolites and organisms, this study compiled a comprehensive large-scale data set describing the seed metabolite sets and metabolite sets of 124,192 pathogenic strains. The enrichment analysis method was used to screen the specific seed metabolites of each species/genus of pathogenic bacteria. Furthermore, we developed the metabolites of pathogenic microorganisms database (MPMdb; http://qyzhanglab.hzau.edu.cn/MPMdb/), which is intuitive and accessible to users with or without bioinformatics expertise. Based on MPMdb, metabolites and seed metabolites of pathogenic microorganisms could be browsed, searched, predicted, and downloaded. In addition, based on the MPMdb data, the taxonomic classification of pathogenic bacteria was performed in terms of the function of seed metabolites and metabolites. The results showed that the seed metabolites could reflect the phylogeny of pathogenic bacteria. The specific seed metabolites of pathogenic bacteria could be used for further tapping the nutritional sources and nutrient deficiency types of pathogenic bacteria and for designing specific bactericide for pathogenic bacteria. Our developed MPMdb will provide a powerful resource platform for the vast number of pathogenic bacterium research projects.

## RESULTS

### The biological importance of seed metabolites of pathogenic microorganisms

Seed metabolites provide important information for the metabolic capacity and growth characteristics of species and reveal the evolution mechanism of species involved in metabolism as well as environmental changes. In addition, a design strategy of specific bactericide based on seed metabolites was proposed in our group, and the developed specific and selective bactericides against *Xanthomonas oryzae* were synthesized and validated, confirming the important role of seed metabolites in *X. oryzae* and the validity of the screening framework for seed metabolites based on metabolic network topology approach.

**Fig 2:**
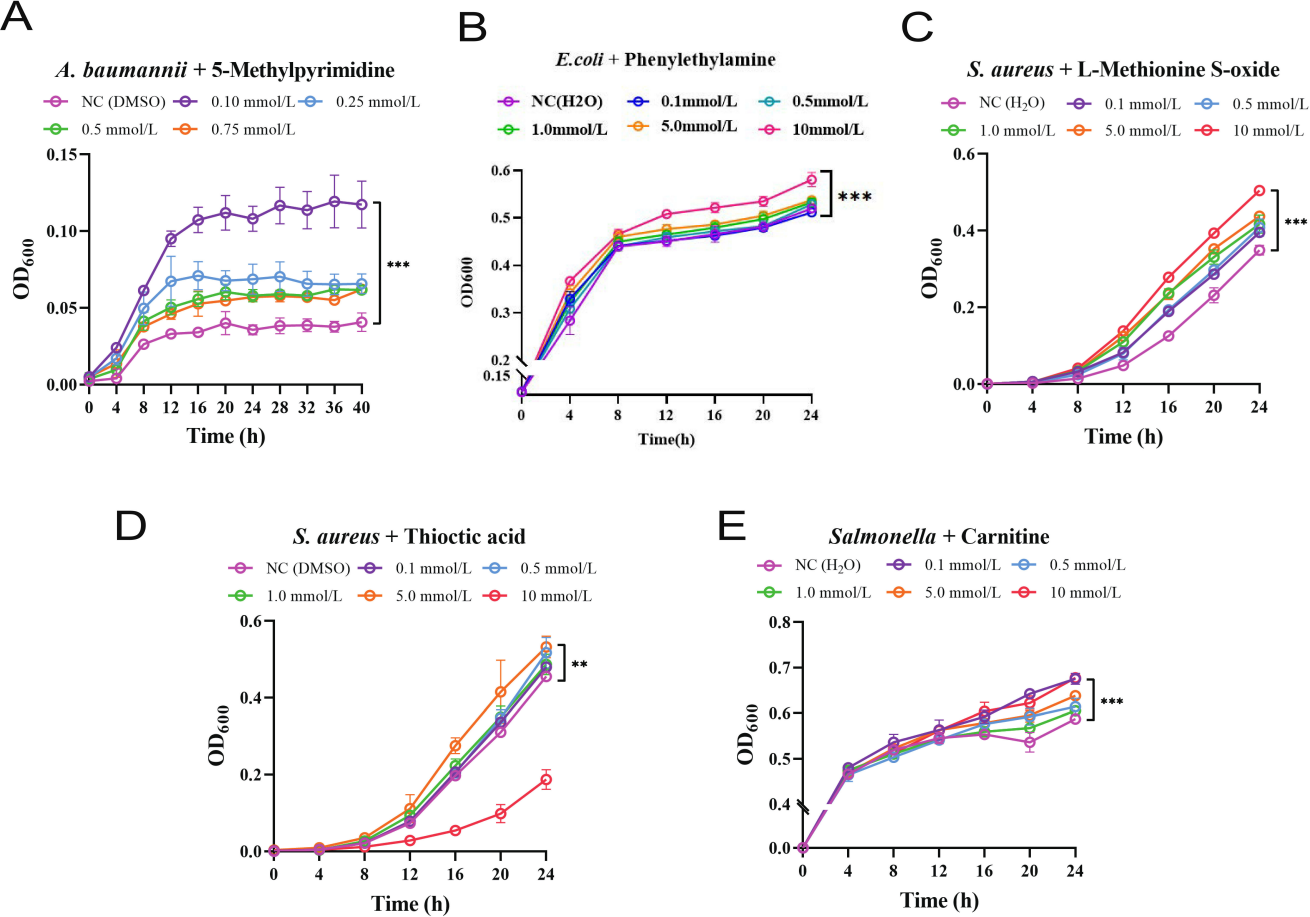
The turbidimetric growth curve of *A. baumannii* at different concentrations of 5-Methylpyrimidine (**A**). The turbidimetric growth curve of *E. coli* at different concentrations of Phenylethylamine (**B**). The turbidimetric growth curve of *S. aureus* at different concentrations of L-Methionine S-oxide (**C**) and Thioctic acid (**D**). The turbidimetric growth curve of *Salmonella* at different concentrations of Carnitine (**E**).

The accuracy of our seed metabolite screening method was validated by selecting combinations of Gram-negative or Gram-positive bacteria with their corresponding seed metabolites, followed by conducting turbidimetric growth curve experiments (Fig. 2). The selection of bacteria and seed metabolites for these validation experiments was based on the specific metabolic needs of various Gram-positive or Gram-negative bacteria and the biological functions of the seed metabolites. For instance, 5-Methylpyrimidine, which could impact nucleotide synthesis, was selected for the multidrug-resistant *Acinetobacter baumannii*; Phenylethylamine, crucial for protein synthesis, was chosen for *Escherichia coli*, a model organism in microbial metabolic research; L-Methionine S-oxide and Thioctic acid, potentially affecting redox reactions, were selected for the highly pathogenic *Staphylococcus aureus*; and finally, Carnitine, key in fatty acid metabolism, was chosen for *Salmonella*. These selections aimed to deeply investigate how metabolic pathways influence bacterial growth, thereby providing key insights into the species-specificity of metabolism and exploring potential antimicrobial targets. In each tested combination, seed metabolites were found to enhance the growth of the corresponding bacteria compared to controls. Notably, linear growth responses were observed in the combinations of *E. coli* with Methionine and *S. aureus* with L-Methionine S-oxide, indicating a direct correlation between seed metabolite concentration and bacterial growth (Fig. 2B and C). For the other three combinations, a specific optimal concentration of seed metabolites was identified, promoting maximum bacterial growth, while concentrations exceeding these optimal levels resulted in growth inhibition. Specifically, the optimal concentration of 5-Methylpyrimidine for *A. baumannii* was determined to be 0.10 mmol/L (Fig. 2A), Thioctic acid for *S. aureus* at 5.0 mmol/L (Fig. 2D), and Carnitine for *Salmonella* at 0.10 mmol/L (Fig. 2E). These findings affirm the crucial role of seed metabolites in promoting the growth of the respective bacterial species.

### Generation of metabolite database of pathogenic microorganism

To explore the metabolic characteristics of pathogenic bacteria, MPMdb was constructed, which was a comprehensive web-based one. It provides detailed information on metabolites and seed metabolites of the 124,192 pathogenic bacteria at different taxonomic levels and the intersection analysis of metabolites between different pathogenic bacterial strains existing in MPMdb.

#### Collection of pathogenic bacteria information and identification of seed metabolites

First, the background information and corresponding whole genome sequence of each pathogenic bacterium were collected from PATRIC (27), VFDB (28), PHI-base (29), and other public databases (30), and the low-quality genomic sequences were manually deleted. The duplicated genome sequences of pathogenic strains with the highest N50 or lowest L50 values were retained. Pathogenic microorganism without clear host information or geographic distribution information was removed from the data set. Finally, a total of 124,192 pathogenic microorganisms belonging to 898 species and 34 genera were screened. Taxonomic classification information of each pathogenic strain was obtained from NCBI (31) and PATRIC (27). The detailed information of metabolites was collected from PubChem (32), Bigg (33), and ChEMBL (34).

**Fig 3:**
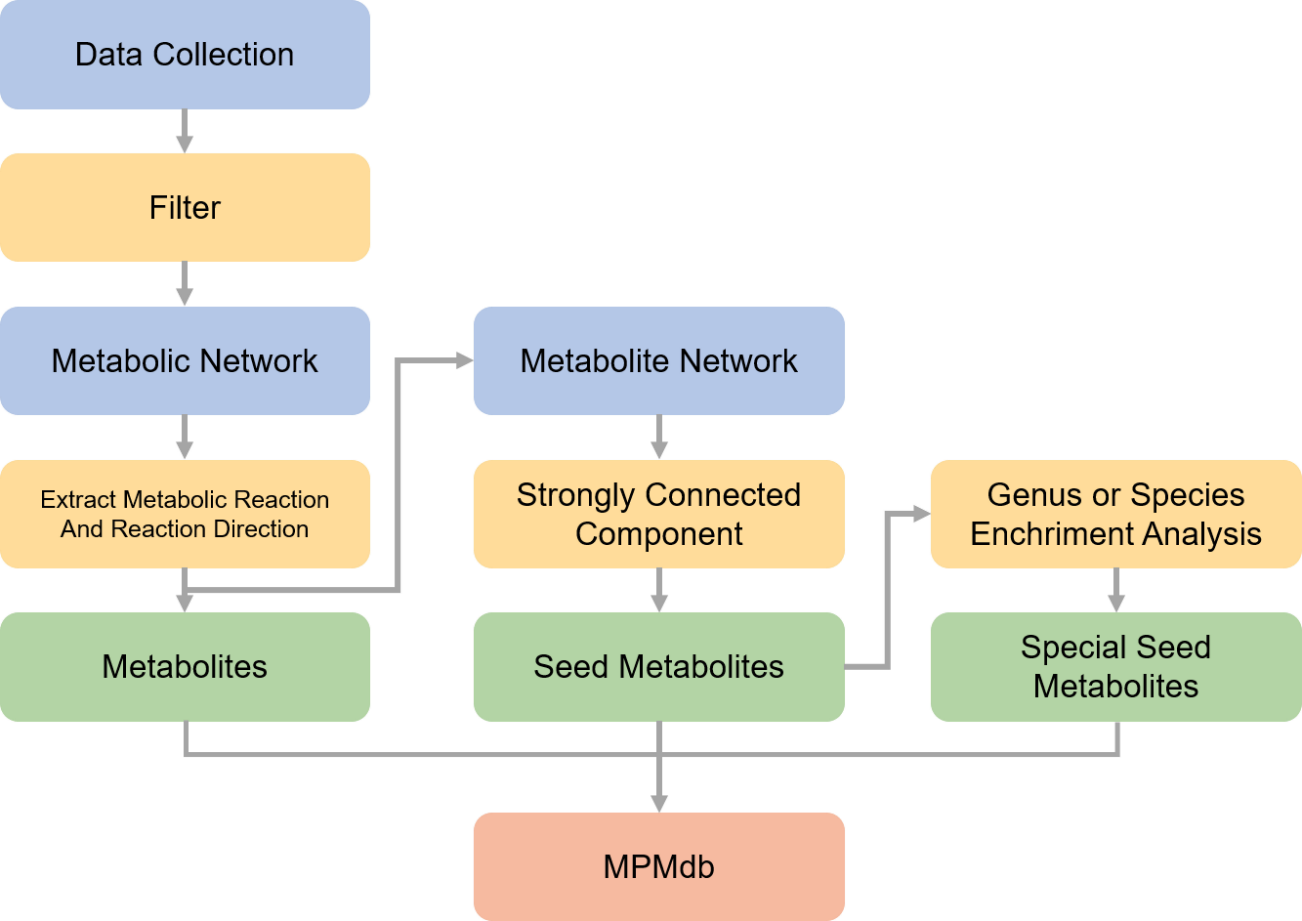
Data processing of MPMdb.

CarveMe is a GSMM reconstruction tool, which uses a top-down approach to build microorganism’s models in a fast and batch manner (35), and the performance of CarveMe models is close to manually curated models in respect of reproducing experimental phenotypes. CarveMe with default parameters was selected to construct the genome-scale metabolism model for each pathogenic bacterium in a batch and efficient manner (35). The GSMM of each pathogen was simplified into a directed graph of the metabolite network. Based on metabolite network topology, seed metabolites were identified using a graph-based algorithm (see Materials and Methods). Finally, 124,192 metabolic networks were constructed, and the metabolites and seed metabolites of each pathogenic bacterium were identified. In addition, metabolites also were classified according to their host, pathogenic strain, and pathogenic strains’ origin. Enrichment analysis was performed to screen the specific seed metabolites at the genus/species level. The data processing of the MPMdb is shown in Fig. 3.

#### Database implementation

MPMdb was built based on the LAMP (Linux + Apache + Mysql + PHP) framework. MPMdb runs on the Apache 2 web server (https://httpd.apache.org/) with MySQL (https://www.mysql.com/) as a database engine, and Python was used for graph drawing.

#### Data summary of MPMdb

**Fig 4:**
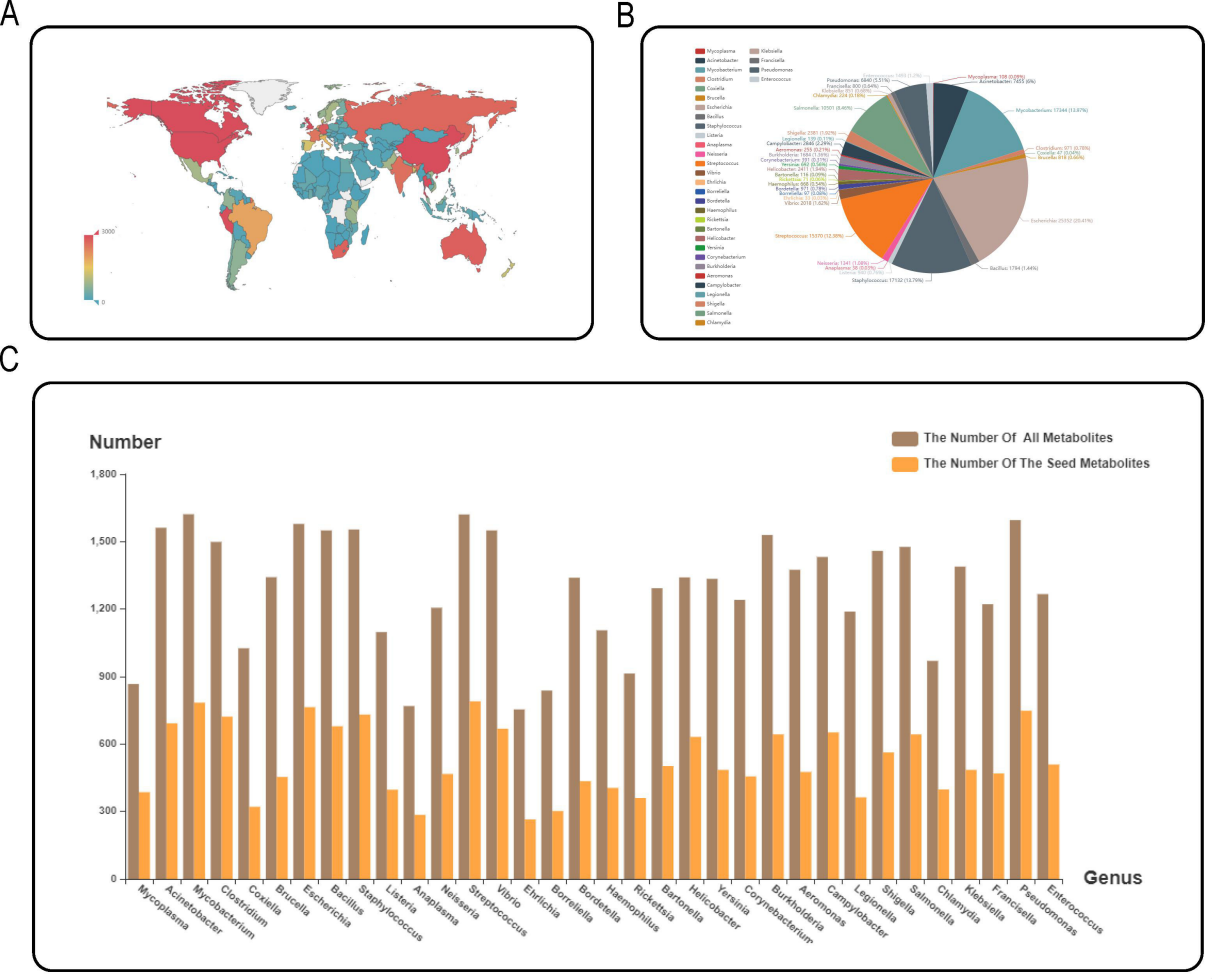
Data summary of MPMdb. (**A**) Global distribution map of pathogenic microorganisms in MPMdb. (**B**) Distribution of pathogenic microorganisms at genus level in MPMdb. (**C**) Number of metabolites and seed metabolites of pathogenic bacterial genera.

The detailed data of statistical information of the built MPMdb data set are summarized and listed in Table S1. The number of species, strains, metabolites, and seed metabolites of each genus was listed in each column of Table S1. All the collected 124,192 pathogenic microorganisms in MPMdb come from 170 regions, and their global distribution map is shown in Fig. 4A. The darker the color, the denser the bacterium distribution. The pathogenic strains of MPMdb come from 34 different genera. The pathogenic strain number of each genus is summarized in Fig. 4B. Different colors represent different genera. The metabolites and seed metabolites from different genera were counted, and the detailed information is shown in Fig. 4C.

#### Web page of MPMdb

**Fig 5:**
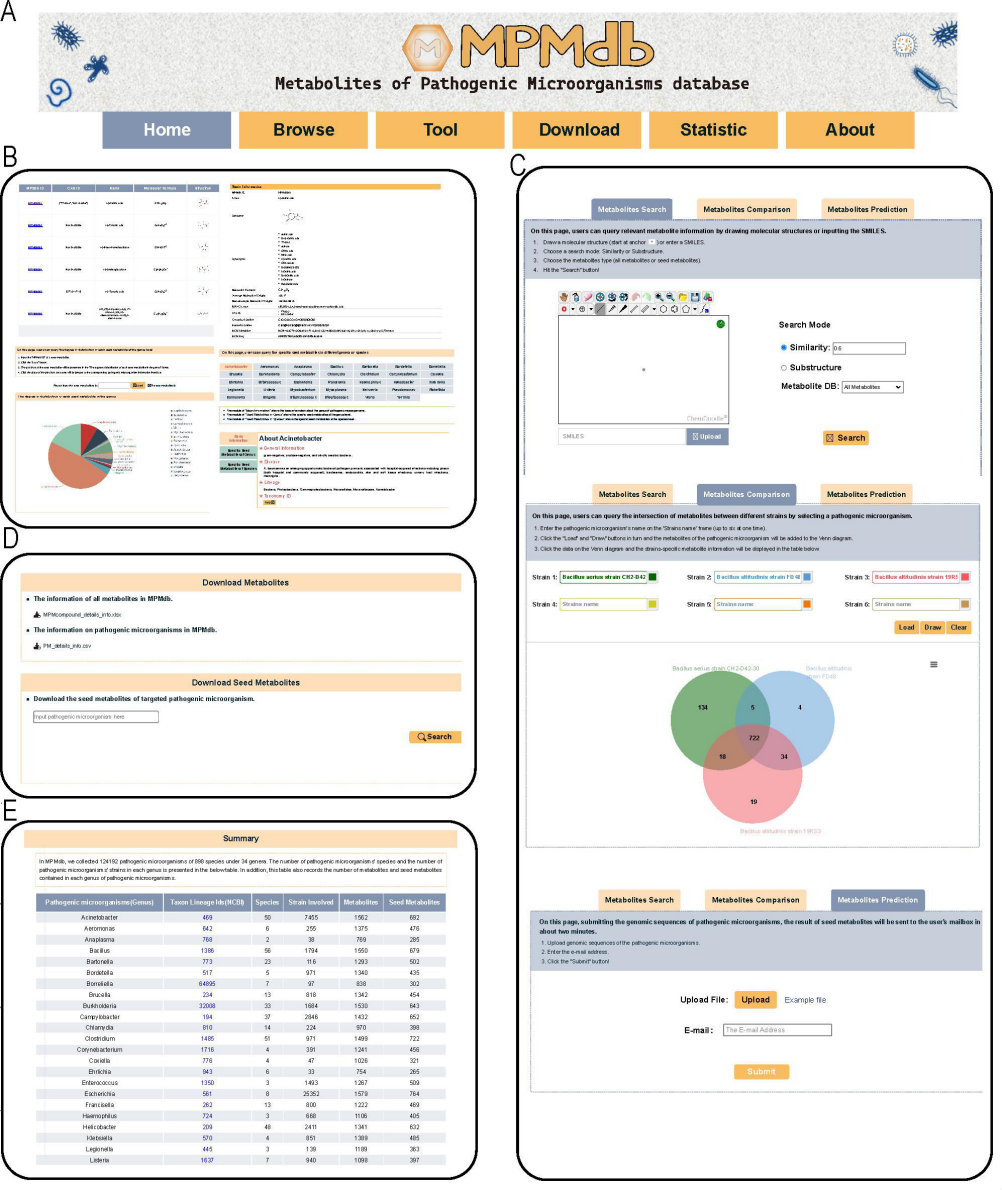
Overview of the MPMdb. (**A**) Six pages of MPMdb including “Home,” “Browse,” “Tool,” “Download,” “Statistic,” and “About.” (**B**) Three modules on “Browse” page including “Metabolites,” “Seed Metabolite Distribution,” and “Specific Seed Metabolites.” (**C**) Three modules on “Tool” page including “Metabolite Search,” “Metabolite Comparison,” and “Metabolite Prediction.” (**D**) “Download” page. (**E**) “Statistics” page.

The built MPMdb provides a user-friendly web interface, as shown in Fig. 5. Six main web pages, including “Home,” “Browse,” “Tool,” “Download,” “Statistic,” and “About,” provide MPMdb introduction for a user to browse metabolites and seed metabolites of pathogenic microorganisms at species/genus levels, to query the distribution of metabolites and seed metabolites at the genus level, to retrieve seed metabolites of pathogenic bacteria of interests, and to download corresponding data sets.

The “Browse” page (Fig. 5B) consists of three modules including “Metabolites,” “Seed Metabolites Distribution,” and “Specific Seed Metabolites.” In the “Metabolites” module, users can search and browse metabolites or seed metabolites filtered by four choices, types of metabolites (default all metabolites), host, region, and pathogenic microorganisms. The users can combine different choices and click “apply” button, and a metabolite table related to certain choices will be generated. Then, by clicking the MPMdb ID of metabolite, the users can view a page presenting detailed metabolite information, especially, the information on the metabolite distribution of pathogenic bacteria at the genus level. In the “Seed Metabolites Distribution” module, users can query the distribution of each metabolite by inputting the seed metabolite name or the MPMdb ID. In the “Specific Seed Metabolites” module, users can browse and query the specific seed metabolites at the genus/species level.

The “Tool” page (Fig. 5C) consists of three modules, including “Metabolite Search,” “Metabolite Comparison,” and “Metabolite Prediction.” In the “Metabolite Search” module, users can query relevant metabolite information by drawing molecular structures or inputting the SMILES file. In the “Metabolite Comparison” module, users can query the intersection of metabolites between different strains by selecting pathogenic microorganisms. In the module of “Metabolite Prediction,” user can receive target seed metabolites through the mailbox in about two minutes after submitting the genomic sequences of customized pathogenic microorganisms.

In addition to data query, users can download the database for data mining via the web interface (Fig. 5D). Bulk downloads of several complete data sets are available at http://qyzhanglab.hzau.edu.cn/MPMdb/Download.php. Furthermore, users can customize the metabolite download list through the advanced search and manual selection on “Browse” page.

On “Statistic” page (Fig. 5E), the detailed data statistics information of the built MPMdb data set is summarized in the form of Tables or Figure.

The “About” page provides the overview, help documentation, and contact information of the MPMdb. The web introduction of MPMdb and the email addresses of the authors are also provided on this page. Any feedback on MPMdb is welcome with the email address attached on the “Contact” page.

### Applications of MPMdb for pathogenic bacteria research

#### Application of MPMdb for taxonomic classification of pathogenic bacteria

The classification of bacteria is important basic research. Traditional classification methods include phenotypic, chemotaxonomic, and genotypic methods (36). Bacteria were classified in terms of phenotypic markers at the beginning of bacterial taxonomy (37). Later on, chemotaxonomic and genotypic methods were widely used as more satisfactory classification approaches than phenotypic one (38). Chemotaxonomic data are very useful and reliable for classification and identification of bacteria. Since the production of secondary metabolites appears to be strain-specific, secondary metabolites are usually used for the chemotaxonomy of microbes (39). However, it is well known that the annotation of secondary metabolite is incomplete. In an early study, Borenstein et al. (17) performed a principal component analysis of the seed metabolites of various taxa (bacteria, plants, animals, archaea, fungi, and protozoan species) and found that seed metabolites are a good characteristic representation of various taxa (17). Therefore, we further explored whether seed metabolites could characterize the genus of each pathogenic microorganism.

A total of 11,500 pathogenic bacterial strains belonging to 23 genera (500 strains per genus) were randomly selected from MPMdb and used as a testing set. The metabolites and seed metabolites of these 11,500 pathogenic bacterial strains were analyzed using the t-distributed Stochastic Neighbor Embedding (t-SNE) algorithm (40) to determine whether these metabolites and seed metabolites could significantly distinguish 23 genera.

**Fig 6:**
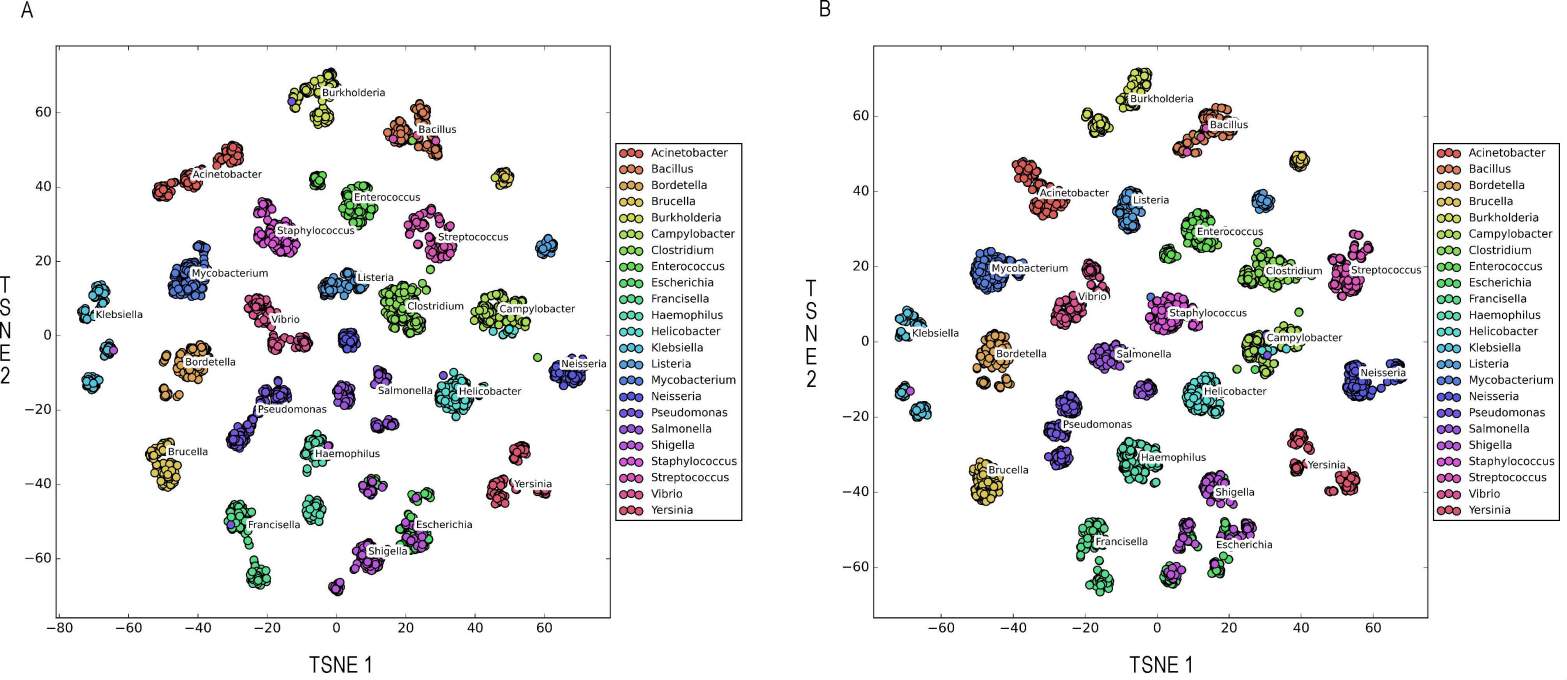
t-SNE analysis of different pathogenic bacterial genera based on metabolites (**A**) and seed metabolites (**B**).

The cluster analysis results are shown in Fig. 6. The t-SNE (40) analysis results showed that most of the metabolites or the seed metabolites of different pathogenic bacterial genera gathered separately except *Shigella* and *E. coli*, indicating that metabolites or seed metabolites could accurately capture the characteristics of different bacterial genera. Previous studies have confirmed that *Shigella* and *E. coli* share many common features in phenotype, and they share 80%–90% identity in genome sequence, whereas other *Escherichia* species are genetically distant (41). Although the number of seed metabolites of pathogenic bacteria is significantly smaller than that of metabolites, they could be used for distinguishing different pathogenic bacterial genera through t-SNE analysis. Thus, the constructed MPMdb will provide valuable basic information for the characterization of each pathogenic species. Our research results further verified that the seed metabolites could be used as a feature for microorganism chemotaxonomy.

#### Application of MPMdb for phylogenetic analysis of pathogenic bacteria

The accurate classification of pathogenic bacteria is an important foundation to explore their phylogeny. Genes are often used to infer accurate species phylogenies. The growth environment of species is closely related to the phylogeny of species. However, genes can only indirectly reflect the growth environment of species. Seed metabolites, as products of the interactions between species and environment, can well reflect the environment. Therefore, seed metabolites provide a novel perspective for the investigation of species evolution.

In order to further explore the characteristics of seed metabolites of pathogenic bacteria, the relationship between seed metabolites and the phylogeny of pathogenic bacteria was examined in our study. Of 898 species under 34 genera, only 323 species had complete genome sequences. Therefore, a pathogenic strain was randomly selected from each species of 323 species (Table S2), and accordingly, its complete genome sequence and 16S rRNA sequence were extracted. Based on 16S rRNA sequences from 323 species and 120 ubiquitous single-copy proteins (bar120) (42) from 323 species, a phylogenetic tree was constructed, respectively. Furthermore, a hierarchical clustering tree was constructed based on the metabolites and seed metabolites of 323 pathogenic strains, respectively. The topologies of all the built trees were compared with each other. The phylogenetic tree based on the bar120 was selected as a standard phylogenetic tree, and the tree based on 16S rRNA was used as a control.

**Fig 7:**
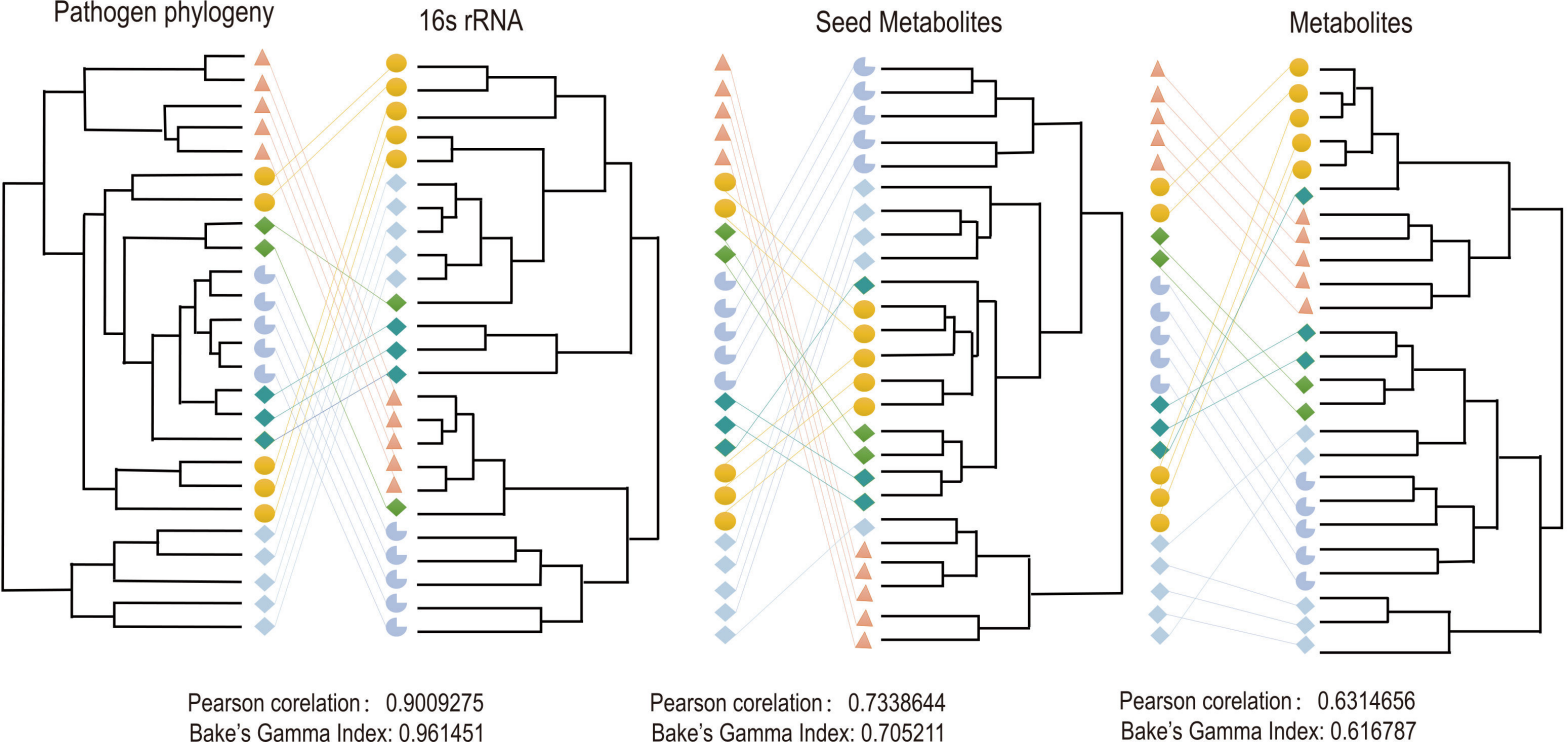
Comparison between bar120 phylogenetic tree and 16S RNA phylogenetic tree, seed metabolite hierarchical clustering tree, or metabolite hierarchical clustering tree.

The results showed that the branching order of the hierarchical clustering tree based on the metabolites and seed metabolites was almost consistent with that of the phylogenetic tree based on the bar120 and 16S rRNA (Fig. 7; Fig. S1). The phylogenetic tree constructed based on bar120 exhibited the highest correlation with that based on 16S rRNA (Pearson correlation  =  0.9009275, Baker’s Gamma Index  =  0.961451, and *P*-value < 0.01), followed by the tree constructed based on seed metabolites (Pearson correlation  =  0.7338644, Baker’s Gamma Index  =  0.7052111, and *P*-value < 0.01), and the lowest correlation with the tree constructed based on metabolites (Pearson correlation  = 0.6314656, Baker’s Gamma Index  = 0.616787, and *P*-value < 0.01). The correlation between the phylogenetic tree based on 16S rRNA and the hierarchical clustering tree based on seed metabolites (Pearson correlation  = 0.6514477, Baker’s Gamma Index  =  0.635633, and *P*-value < 0.01) was similar to the correlation between the phylogenetic tree based on 16S rRNA and the hierarchical clustering tree based on metabolites (Pearson correlation  =  0.6485358, Baker’s Gamma Index  =  0.619793, and *P*-value < 0.01).

The correlation between bar120 tree and seed metabolites tree was not as high as the correlation between bar120 tree and the 16S rRNA tree, which might be because 16S rRNA served as a phylogenetic classification index. The closer the species determined by seed metabolites, the closer its corresponding evolutionary distance. These results indicate that the composition of seed metabolites of pathogenic bacteria reflected better phylogeny of pathogenic bacteria than the composition of metabolites of pathogenic bacteria. Therefore, a smaller number of seed metabolites could contain more valid information, indicating that MPMdb could mirror the phylogeny of pathogenic bacteria by seed metabolites to some extent.

#### Application of MPMdb for nutrient requirement analysis of pathogenic bacteria

All microbes rely on other species or environment to provide specific nutrients that cannot be produced by themselves. These nutrients refer to unique nutritional components and auxotrophies of microbes. Flux-balance analysis (FBA) (43) is usually used to analyze organisms’ nutrient requirements. Since FBA is based on the understanding of the growth needs and biomass of organisms, it cannot be used for analyzing those uncultured microorganisms. Our previous study has proposed the concept of specific seed metabolites of pathogenic bacteria of different genus/species. Specific seed metabolites refer to unique and specific seed compounds identified from pathogenic bacteria by enrichment analysis. Specific seed metabolites could offer a potentially effective approach to analyze the unique nutritional components and auxotrophies of pathogenic bacteria.

In this study, *Streptococcus mutans* was selected for nutrient requirement analysis. *S. mutans* is a Gram-positive bacterium that thrives under acidic conditions and is a primary cause of tooth decay (dental caries) (44). In the oral cavity, competitive nutrition acquisition is crucial for bacterial normal growth. *S. mutans* can thrive in the oral environment. One of the important reasons is the flexibility of their metabolism, and *S. mutans* develops multiple strategies to absorb carbon sources and necessary biomacromolecules from the oral environment.

Based on the seed metabolites information of pathogenic bacteria provided in the MPMdb, key metabolites, such as thiamine, riboflavin, NAD+/NADP+, pantothenic acid, folic acid, and glutathione, were captured. These captured compounds have been reported to be unique metabolites that could not be synthesized by *S. mutans*, and thus, *S. mutans* has to obtain them from the environment to sustain its growth (44). Therefore, the captured compounds are considered to be potential auxotrophies of *S. mutans*. In addition, *S. mutans* is also auxotrophic for glutathione, and the glutathione acquisition from the host is crucial to the *S. mutans* antioxidant defense system, which facilitates the escape from the host innate immune response (45).

The above findings indicated that MPMdb was helpful for capturing the unique metabolites of pathogenic bacteria, thus improving nutrient prediction based on seed metabolites of each pathogen.

## DISCUSSION

Seed metabolites are a combination of essential compounds required by pathogenic bacteria across various potential environmental conditions, crucial for their growth, reproduction, external communication, and host infection. The MPMdb developed in this study systematically predicts and identifies the metabolites and seed metabolites of pathogenic microorganisms, aiming to resolve the issues of low throughput and high costs in large-scale screening, thus providing a valuable resource for pathogenic bacteria research, with significant research implications. Here, the feasibility of the screening framework and the effectiveness of the seed metabolites were executed and confirmed by experiment validation. Then, we have constructed MPMdb, a user-friendly resource for the exploration of the metabolic characteristics of pathogenic bacteria. MPMdb provides a flexible search interface, detailed information about metabolites of pathogenic bacteria, and convenient tools, thus allowing users to identify and screen the certain metabolites of each species/genus of pathogenic bacteria. Meanwhile, the functions of MPMdb were further explored. MPMdb can be used for exploring the relationship between seed metabolites and pathogen characteristics, pathogen phylogeny, or the nutrients requirement of pathogen. In addition, the roles of seed metabolites from MPMdb in designing specific and selective bactericides were further confirmed. This work provides new perspectives for pathogenic bacteria research.

Our analysis is based on metabolic networks constructed through automated modeling. While the structural complexity of pathogenic bacteria is relatively simpler compared to eukaryotes, the predictions of seed metabolites in these automatically constructed networks align closely with those from manually curated networks (46). However, the potential for false positives remains due to gaps in the metabolic network. The strength of our approach lies in its ability to construct metabolic networks on a large scale, enabling the batch identification of seed metabolites in pathogenic bacteria. Furthermore, the application of hypergeometric testing effectively reduces the occurrence of false positives, enhancing the reliability of our findings in investigating the infection mechanisms of microorganisms.

Several seed metabolite-based tools have been used for elucidating microbial metabolism and ecology, such as NetSeed (47) and NetCooperate (48), which have been developed for studying species interactions based on seed metabolite strategies. While NetSeed and NetCoordinate offer valuable insights, they do require species-specific metabolic models and are primarily geared toward small-scale analysis. PhyloMint (49) has predicted complementary and competitive metabolic relationships between bacterial species based on seed metabolite strategies. The aim of PhyloMint is to calculate competition and complementarity indices for pairs of microbial species. This approach, while innovative, presents certain challenges for users in directly accessing the seed sets of these species. RevEcoR (50) supports the reconstruction of species metabolism models using the annotated metabolic data, although acquiring such data requires additional resources.

Compared with previous studies (47–50), our work aims to explore the relationship between seed metabolites and pathogenic bacteria by comprehensively analyzing the massive data sets obtained by the seed metabolite screening algorithm. In addition, specific seed metabolites can not only be used for the taxonomic classification of pathogenic bacteria but also provide a new idea for the design of specific bactericide. By integrating the unique seed metabolites of pathogenic bacteria with existing bactericides, we can design more specific bactericidal agents. This approach holds the potential to enhance bactericidal efficiency and effectively address the current challenges of drug resistance.

Future work can be devoted to further the improvement of seed metabolite screening algorithm and including experimental data into MPMdb so as to further expand these metabolic resources of pathogenic bacteria. Additionally, we will further refine the metabolic model of each pathogen to further reveal the relationship between seed metabolites and pathogens, clarify the functions of seed metabolites in drug development, pathogen classification, and pathogenicity mechanism research, and embed these relating parts into the database as modules.

## MATERIALS AND METHODS

### Data collection and preprocessing

The genomic sequences of pathogenic microorganisms were collected from PATRIC (27), VFDB (28), PHI-base (29), and other public databases (30). The collected sequences were subjected to the following preprocessing. First, high-quality genomic sequences of pathogenic bacteria (as defined by PATRIC, which uses CheckM to assess genome quality) from the module of “Bacterial Pathogens” in the PATRIC database were collected. Meanwhile, all pathogenic bacterial genomes from VFDB and PHI-base were collected. Second, for duplicated genome sequences of all collected pathogenic strains, only the versions with the highest N50 value or the lowest L50 value were retained (51). Finally, pathogenic microorganism genome sequences with clear host information through manually querying their records in the source databases were selected for further research. The genome sequences of pathogenic microorganisms were classified according to lineages of pathogenic microorganisms, and a total of 124,192 whole genome protein sequences of pathogenic microorganisms belonging to 34 genera were collected.

### Construction of organism-specific directed network based on metabolic model

In our study, CarveMe (35) was employed with its standard default settings to systematically construct the metabolic networks of various pathogenic bacteria. CarveMe automates the creation of GSMMs by first inputting genomic sequences of target organisms to generate draft SBML models. These models are then refined by removing nonpresent metabolic reactions and compounds, resulting in tailored, organism-specific GSMMs that accurately reflect the metabolic capabilities inferred from the genomic data. The metabolic network of bacteria was primarily read using the cobra.io.read_sbml_model function from COBRApy (52). The reactions were subsequently extracted from the network, and the nx.DiGraph function from NetworkX was utilized to construct a directed graph. Directed edges were added using the add_edge function. Finally, the directed metabolite network of the metabolic model of each pathogenic microorganism was constructed, and the obtained each directed metabolite network was composed of multiple nodes and edges with nodes representing metabolites and edges representing the corresponding metabolic reactions as well as their directions. The metabolites of each pathogenic microorganism were determined by extracting all the nodes in directed network of metabolic model.

### Prediction of metabolites and seed metabolites of pathogenic bacteria

The seed metabolites of each pathogenic microorganism were acquired by strongly connected component (SCC) algorithm method (53). SCC is a maximal node set in which there is at least one path connecting these two nodes of each node pair. The SCC forms a directed acyclic graph where nodes are the components and edges are the original edges connecting nodes representing two different components. The organism-specific directed network was decomposed into SCCs using the nx.strongly_connected_components function.

The identification of seed metabolites involved identifying SCCs that had no in-degree but at least one out-degree among all SCCs, which were then defined as “source components.” Metabolites belonging to all source components were considered as candidate seed metabolites. To ensure biological relevance, this set was further constrained by considering only those metabolites whose source component scale (i.e., the number of metabolites contained) was not greater than 5 (17).

### Screening of specific seed metabolites

The specific seed metabolites were designated as the seed metabolites significantly enriched in a certain sample. In MPMdb, we defined the sample as genus/species. The screened seed metabolites were subjected to a hypergeometric test to determine whether a seed metabolite was significantly enriched in a certain sample. The seed metabolites with *P*-value ≤ 0.01 were defined as the specific seed metabolites.

The hypergeometric test formula is as follows (54):


p≥j=∑i=j∞KiM-KN-iMn

Where *N* indicates the number of pathogen strains in MPMdb (124,192); *M* represents the strain number in a given species sample; *i* indicates the strain number containing a certain seed metabolite among 124,192 pathogen strains; *K* indicates pathogen strain number that contains a certain seed metabolite among the given strain sample; *j* indicates the strain number containing a certain seed metabolite observed in the experiment. Here, setting *j* to one indicates that there is at least one pathogen strain that contains the specific seed metabolite.

### Hierarchical clustering analysis

Hierarchical clustering trees of metabolites and seed metabolites were constructed using the hclust function and analyzed using the packages dendextend v1.16.0 (55) and ape v5.6.2 (56) (Jaccard distances and Ward’s D2 clustering method), respectively. The correlation between trees was analyzed in terms of Pearson correlation coefficients and Baker’s Gamma Index in the dendextend package (57).

### Phylogenetic analysis of 120 ubiquitous single-copy proteins (bar120) and 16S rRNA

To build the phylogenic tree based on 16S rRNA genes, the substitution model was determined using MEGA 7 (58). The phylogenetic tree was constructed by the maximum likelihood method using MEGA 7 and the substitution model GTR + G + I.

The “120 ubiquitous single-copy proteins” are usually used for bacterial classification as marker genes (42). First, the gtdbtk identified and gtdbtk align workflows in Genome Taxonomy Database toolkit version 2.1.1 (GTDB-Tk) (59) were employed to identify and concatenate 120 ubiquitous single-copy bacterial marker genes of 323 pathogenic strains. The substitution model was determined using MEGA 7. The maximum likelihood phylogenetic tree was established using IQ-TREE 2.0.3 program (60), SH-aLRT test, 1,000 repeated ultrafast guidance, and LG + G + I model.

### Turbidimetric growth curve experiment of seed metabolites

To verify the importance of the screened seed metabolites for the growth of common pathogenic microorganisms, turbidimetric growth curve experiment was performed by measuring the optical density at 600 nm after 0, 4, 8, 12, 16, 20, and 24 h post the addition of seed metabolites. The M9 medium whose composition was continuously adjusted based on the properties of pathogenic bacteria was utilized as minimal medium, and pathogenic microorganisms’ strains were grown in the liquid Medium at 28°C. The composition of adjusted M9 medium of *S. aureus*, *Salmonella*, and *E. coli* is as follows.

*S. aureus*: C6H12O6 (glucose; 2 g), MgSO4 (0.24 g), CaCl2 (0.011 g), KH2PO4 (3 g), Na2HPO4 (6.78 g), NaCl (0.5 g), NH4Cl (1 g; pH = 7.0).

*Salmonella* and *E. coli*: C6H12O6 (glucose; 0.5 g), MgSO4 (0.24 g), CaCl2 (0.011 g), KH2PO4 (3 g), Na2HPO4 (6.78 g), NaCl (0.5 g), NH4Cl (0.25 g; pH = 7.0).

The seed metabolites were dissolved in dimethyl sulfoxide (DMSO) whose volume accounted for no more than 0.2% of the medium volume. The concentration gradients of exogenous metabolites in DMSO solvent were set as 0, 0.1, 0.5, 1, 5, and 10 mM. Each concentration was assayed in triplicates. A turbidimetric growth curve was constructed, and the optical density at 600 nm was measured after 0, 4, 8, 12, 16, 20, and 24 h post the addition of seed metabolites. The slope of each growth curve was determined.

## Data Availability

All genomic data used in this study are openly available in public databases (https://www.bv-brc.org/, http://www.mgc.ac.cn/VFs/, http://www.phi-base.org/, http://www.ncbi.nlm.nih.gov/refseq/). Corresponding genomic information and links can be found on the download page of MPMdb (http://qyzhanglab.hzau.edu.cn/MPMdb/Download.php). All processed data generated for this study are included in MPMdb and supplementary materials. However, due to legal and ethical considerations, as well as the ongoing need for further research and potential future publication, wet-lab experimental data are not publicly available.

## SUPPLEMENTAL MATERIAL

Tables S1 to S2，Figure S1 spectrum.02342-23-s0001.pdf Table S1 (Data summary of pathogenic microorganisms), Table S2 (Pathogenic strains pbid in Fig. 6), and Fig. S1 (Tanglegrams with all samples to compare between trees of the data sets [Bray- Curtis dissimilarity and Ward's hierarchical clustering method]: [A] bar120 vs. 16S rRNA; [B] bar120 vs. metabolites; [C] bar120 vs. seed metabolites; [D] 16srrna vs. metabolites; [E] 16srrna vs. seed metabolites; [F] seed metabolites vs. metabolites).

ASM does not own the copyrights to Supplemental Material that may be linked to, or accessed through, an article. The authors have granted ASM a non-exclusive, world-wide license to publish the Supplemental Material files. Please contact the corresponding author directly for reuse.
