# Supplementary material for: Metabolites of pathogenic microorganisms database (MPMdb) and its seed metabolite applications
Source: Microbiol Spectr. 2024 Feb 23;12(4):e02342-23. doi: 10.1128/spectrum.02342-23 (PMC10986615; doi:10.1128/spectrum.02342-23)
Supplement: Tables S1 to S2，Figure S1 — Table S1 (Data summary of pathogenic microorganisms), Table S2 (Pathogenic strains pbid in Fig. 6), and Fig. S1 (Tanglegrams with all samples to compare between trees of the data sets [Bray- Curtis dissimilarity and Ward's hierarchical clustering method]: [A] bar120 vs. 16S rRNA; [B] bar120 vs. metabolites; [C] bar120 vs. seed metabolites; [D] 16srrna vs. metabolites; [E] 16srrna vs. seed metabolites; [F] seed metabolites vs. metabolites). [file spectrum.02342-23-s0001.pdf]

Supplementary information for

Metabolites of Pathogenic Microorganisms database (MPMdb) and its seed metabolite applications

Feng Jiang†, Yao Ruan†, Xiao-Hui Chen†, Hai-Long Yu, Ting Cheng, Xin-Ya Duan, Yan-Guang Liu, Hong-Yu Zhang, Qing-Ye Zhang*
Hubei Key Laboratory of Agricultural Bioinformatics, College of Informatics, Huazhong Agricultural University, Wuhan 430070, P. R. China
*Corresponding author: Qing-Ye Zhang (E-mail: zqy@mail.hzau.edu.cn); Tel: +86-27-8728-0877
† The first three authors contributed equally.

This PDF file includes:
Tables S1 to S2
Figure S1

Table S1. Data summary of pathogenic microorganisms

Pathogenic microorganisms (Genus)	Taxon Lineage ID (NCBI)	Species	Strain Involved	Metabolites	Seed Metabolites
Acinetobacter	469	50	7455	1562	692
Aeromonas	642	6	255	1375	476
Anaplasma	768	2	38	769	285
Bacillus	1386	56	1794	1550	679
Bartonella	773	23	116	1293	502
Bordetella	517	5	971	1340	435
Borreliella	64895	7	97	838	302
Brucella	234	13	818	1342	454
Burkholderia	32008	33	1684	1530	643
Campylobacter	194	37	2846	1432	652
Chlamydia	810	14	224	970	398
Clostridium	1485	51	971	1499	722
Corynebacterium	1716	4	391	1241	456
Coxiella	776	4	47	1026	321
Ehrlichia	943	6	33	754	265
Enterococcus	1350	3	1493	1267	509
Escherichia	561	8	25352	1579	764
Francisella	262	13	800	1222	469
Haemophilus	724	3	668	1106	405
Helicobacter	209	48	2411	1341	632
Klebsiella	570	4	851	1389	485
Legionella	445	3	139	1189	363
Listeria	1637	7	940	1098	397
Mycobacterium	1763	61	17344	1622	784
Mycoplasma	2093	10	108	867	386
Neisseria	482	2	1341	1206	467
Pseudomonas	286	135	6840	1596	748
Rickettsia	780	39	71	914	360
Salmonella	590	4	10501	1477	643
Shigella	620	7	2381	1459	563
Staphylococcus	1279	56	17132	1554	731
Streptococcus	1301	98	15370	1621	790
Vibrio	662	69	2018	1550	668
Yersinia	629	17	692	1335	485

Table S2. **Pathogenic strains pbid in Fig.6**

#**PB_id** could be find in MPMdb.
#A hierarchical clustering tree was constructed based on the metabolites and seed metabolites of those 323 pathogenic strains, respectively.
#A phylogenetic tree was constructed based on the bar120 and 16sRNA of those 323 pathogenic strains, respectively.

**PB_id**
PB0069302
PB0121494
PB0121705
PB0121501
PB0121653
PB0123038
PB0122945
PB0123242
PB0121546
PB0123355
PB0123299
PB0021002
PB0086812
PB0041992
PB0088658
PB0087045
PB0044392
PB0044401
PB0044398
PB0044399
PB0077810
PB0079732
PB0086378
PB0086545
PB0086548
PB0079248
PB0086382
PB0049061
PB0049101
PB0049229
PB0049224
PB0123541
PB0123874
PB0123888

PB0123589
PB0045827
PB0045972
PB0007624
PB0007633
PB0007627
PB0007471
PB0007651
PB0007630
PB0009543
PB0009544
PB0009547
PB0009563
PB0010742
PB0010831
PB0011336
PB0011182
PB0010852
PB0007739
PB0046156
PB0046192
PB0048106
PB0048348
PB0047748
PB0048104
PB0048105
PB0048108
PB0046177
PB0048208
PB0013316
PB0014586
PB0015881
PB0015947
PB0015873
PB0015942
PB0015626
PB0015918
PB0013367
PB0015548
PB0015348
PB0015533
PB0015535
PB0099928
PB0105109

PB0105143
PB0104066
PB0105716
PB0105588
PB0105496
PB0105816
PB0105585
PB0105681
PB0105519
PB0105520
PB0105558
PB0105543
PB0105589
PB0105887
PB0105536
PB0104110
PB0100100
PB0100172
PB0104986
PB0100305
PB0100333
PB0104055
PB0104661
PB0104752
PB0104854
PB0104744
PB0104769
PB0008364
PB0009460
PB0008397
PB0009461
PB0009198
PB0008387
PB0009157
PB0008877
PB0009003
PB0009191
PB0009033
PB0009100
PB0008476
PB0009451
PB0009455
PB0009174
PB0009359

PB0009534
PB0009372
PB0009349
PB0009340
PB0009368
PB0009342
PB0009430
PB0009182
PB0008774
PB0050163
PB0050213
PB0050182
PB0050331
PB0050385
PB0106682
PB0118970
PB0120487
PB0120466
PB0121169
PB0120874
PB0120918
PB0120876
PB0121187
PB0118689
PB0118698
PB0118769
PB0118807
PB0121131
PB0119057
PB0121457
PB0120242
PB0121059
PB0121048
PB0121098
PB0121049
PB0121057
PB0121058
PB0121050
PB0119381
PB0121141
PB0108235
PB0120825
PB0017947
PB0019069

PB0016356
PB0016366
PB0016547
PB0016600
PB0016770
PB0016910
PB0017064
PB0017448
PB0017550
PB0017590
PB0017468
PB0017591
PB0017612
PB0060801
PB0067447
PB0067496
PB0067160
PB0067176
PB0067284
PB0067500
PB0067183
PB0067193
PB0067201
PB0067268
PB0067161
PB0067180
PB0067486
PB0067489
PB0067440
PB0067446
PB0067461
PB0067622
PB0067587
PB0067589
PB0067464
PB0067465
PB0016118
PB0016138
PB0016238
PB0016240
PB0016246
PB0016253
PB0016254
PB0016147

PB0016214
PB0016216
PB0016215
PB0016154
PB0016196
PB0016176
PB0016186
PB0016190
PB0016255
PB0010635
PB0011556
PB0011569
PB0011587
PB0011614
PB0011613
PB0011608
PB0012264
PB0012454
PB0012330
PB0011630
PB0011677
PB0012375
PB0012466
PB0012509
PB0013115
PB0013132
PB0012468
PB0012797
PB0010588
PB0010603
PB0009746
PB0010493
PB0010523
PB0010540
PB0010604
PB0068900
PB0044831
PB0049425
PB0000045
PB0000050
PB0001116
PB0001374
PB0001375
PB0000202

PB0001365
PB0001371
PB0001413
PB0001472
PB0000929
PB0001122
PB0007357
PB0007388
PB0000160
PB0003436
PB0001060
PB0007216
PB0007230
PB0007300
PB0000939
PB0001489
PB0001442
PB0000168
PB0001521
PB0001101
PB0075782
PB0069526
PB0075019
PB0075020
PB0069818
PB0069791
PB0075742
PB0075759
PB0069679
PB0075834
PB0075837
PB0075842
PB0075843
PB0075851
PB0075845
PB0075847
PB0075830
PB0075825
PB0075675
PB0075676
PB0075677
PB0075678
PB0075874
PB0075875

PB0069688
PB0075521
PB0075767
PB0075769
PB0075876
PB0075877
PB0075878
PB0075880
PB0075800
PB0069691
PB0069704
PB0075734
PB0075582
PB0075604
PB0075605
PB0075606
PB0069454
PB0075389
PB0075466
PB0075746
PB0075810
PB0075806
PB0075540
PB0075871
PB0075730

A

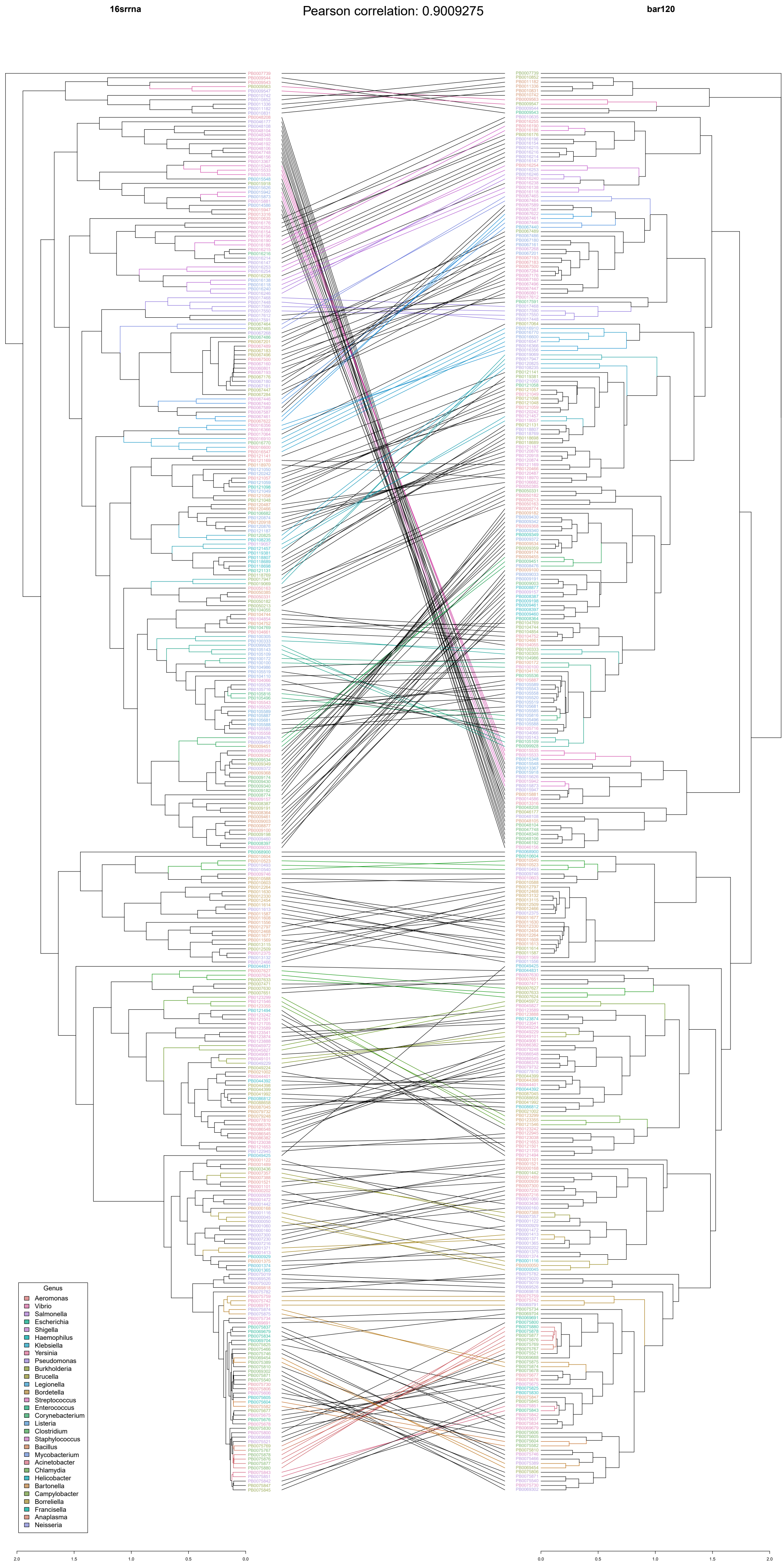
16srrna
Pearson correlation: 0.9009275
bar120
Genus
Aeromonas
Vibrio
Salmonella
Escherichia
Shigella
Haemophilus
Klebsiella
Yersinia
Pseudomonas
Burkholderia
Brucella
Legionella
Bordetella
Streptococcus
Enterococcus
Corynebacterium
Listeria
Clostridium
Staphylococcus
Bacillus
Mycobacterium
Acinetobacter
Chlamydia
Helicobacter
Bartonella
Campylobacter
Borreliella
Francisella
Anaplasma
Neisseria
2.0
1.5
1.0
0.5
0.0
0.0
0.5
1.0
1.5
2.0

B

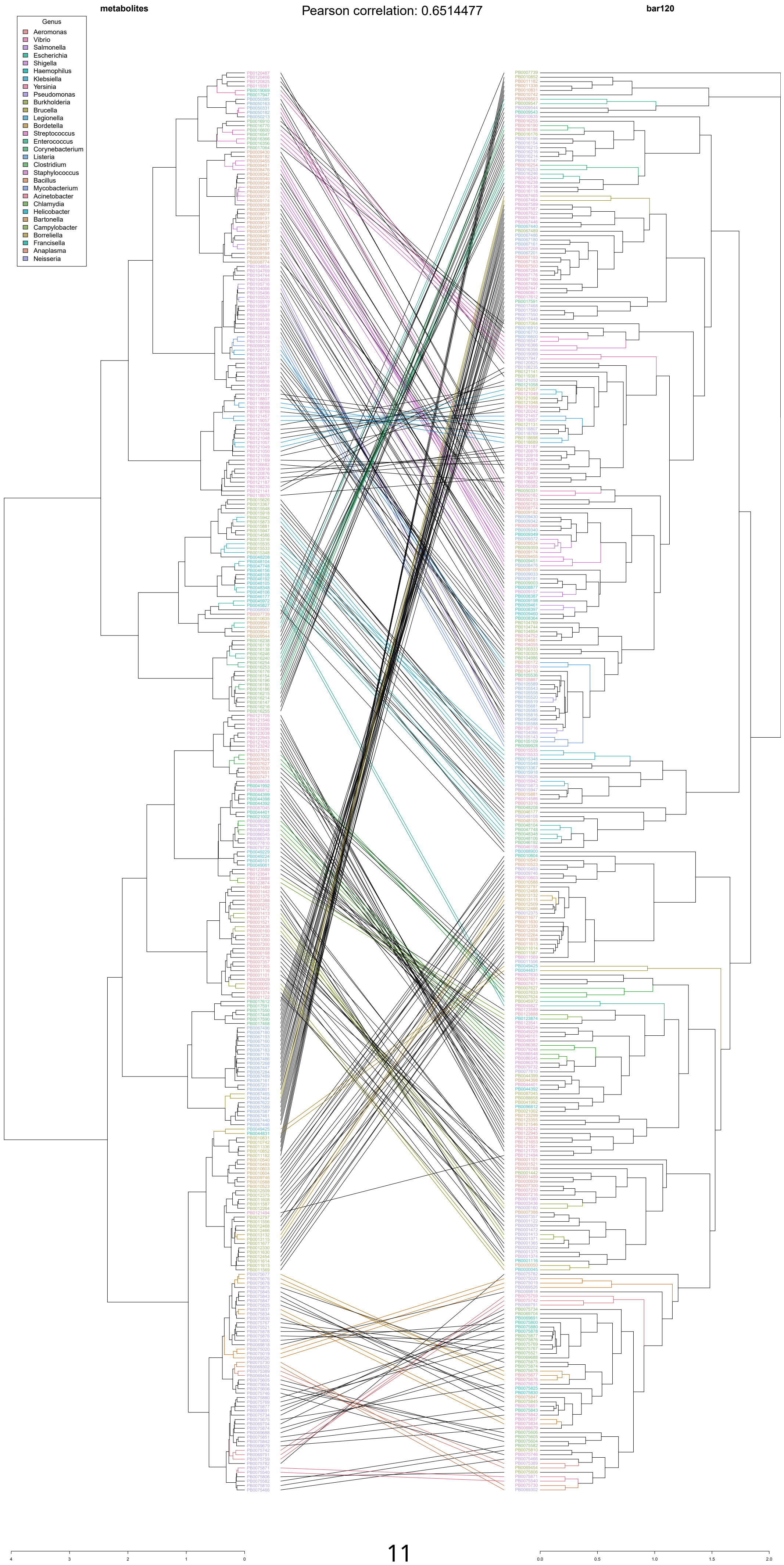
metabolites
Pearson correlation: 0.6514477
bar120
Genus
Aeromonas
Vibrio
Salmonella
Escherichia
Shigella
Haemophilus
Klebsiella
Yersinia
Pseudomonas
Burkholderia
Brucella
Legionella
Bordetella
Streptococcus
Enterococcus
Corynebacterium
Listeria
Clostridium
Staphylococcus
Bacillus
Mycobacterium
Acinetobacter
Chlamydia
Helicobacter
Bartonella
Campylobacter
Borreliella
Francisella
Anaplasma
Neisseria
4
3
2
1
0
0.0
0.5
1.0
1.5
2.0

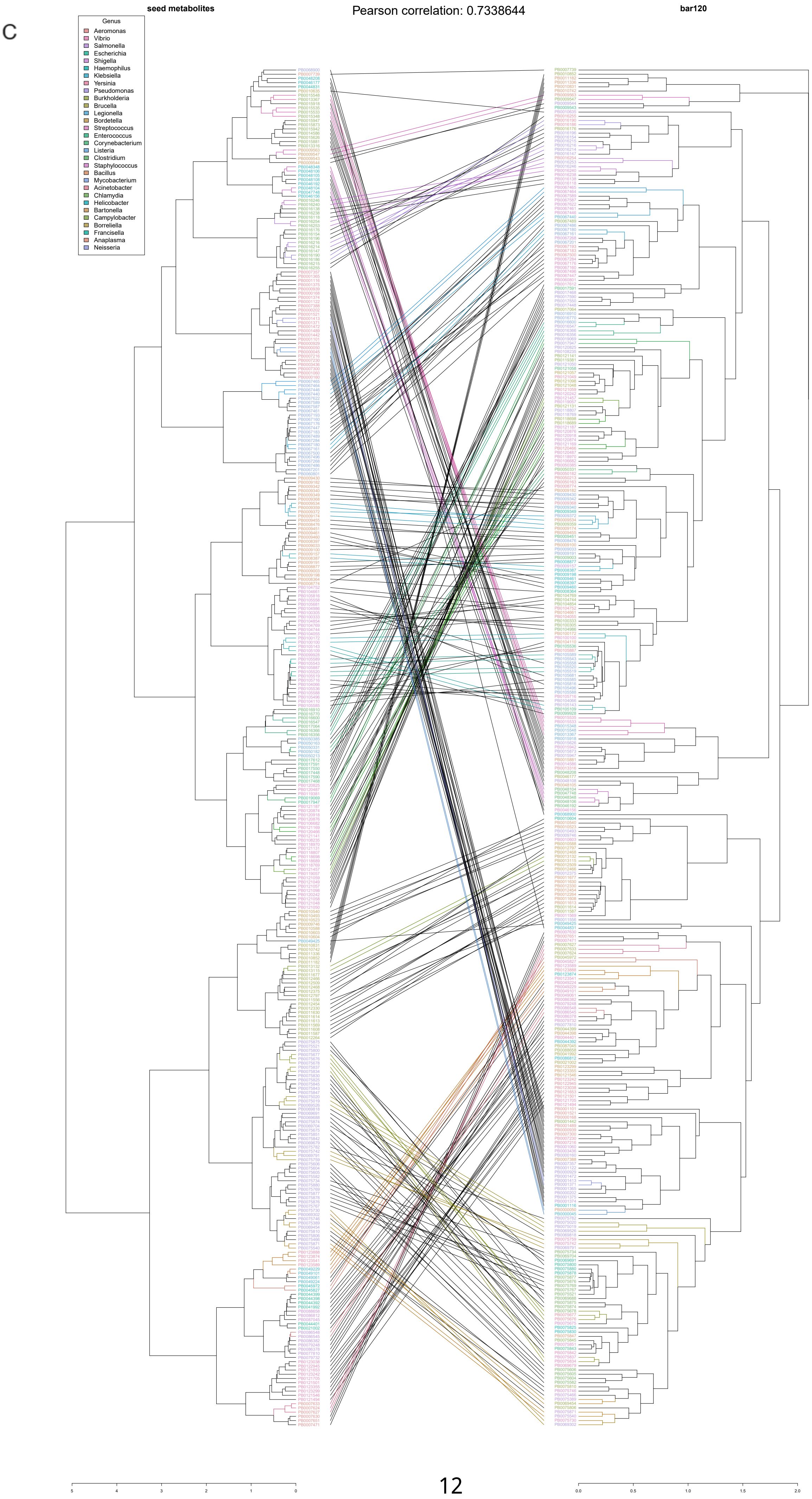
C
seed metabolites
Pearson correlation: 0.7338644
bar120
Genus
Aeromonas
Vibrio
Salmonella
Escherichia
Shigella
Haemophilus
Klebsiella
Yersinia
Pseudomonas
Burkholderia
Brucella
Legionella
Bordetella
Streptococcus
Enterococcus
Corynebacterium
Listeria
Clostridium
Staphylococcus
Bacillus
Mycobacterium
Acinetobacter
Chlamydia
Helicobacter
Bartonella
Campylobacter
Borreliella
Francisella
Anaplasma
Neisseria
5
4
3
2
1
0
0.0
0.5
1.0
1.5
2.0

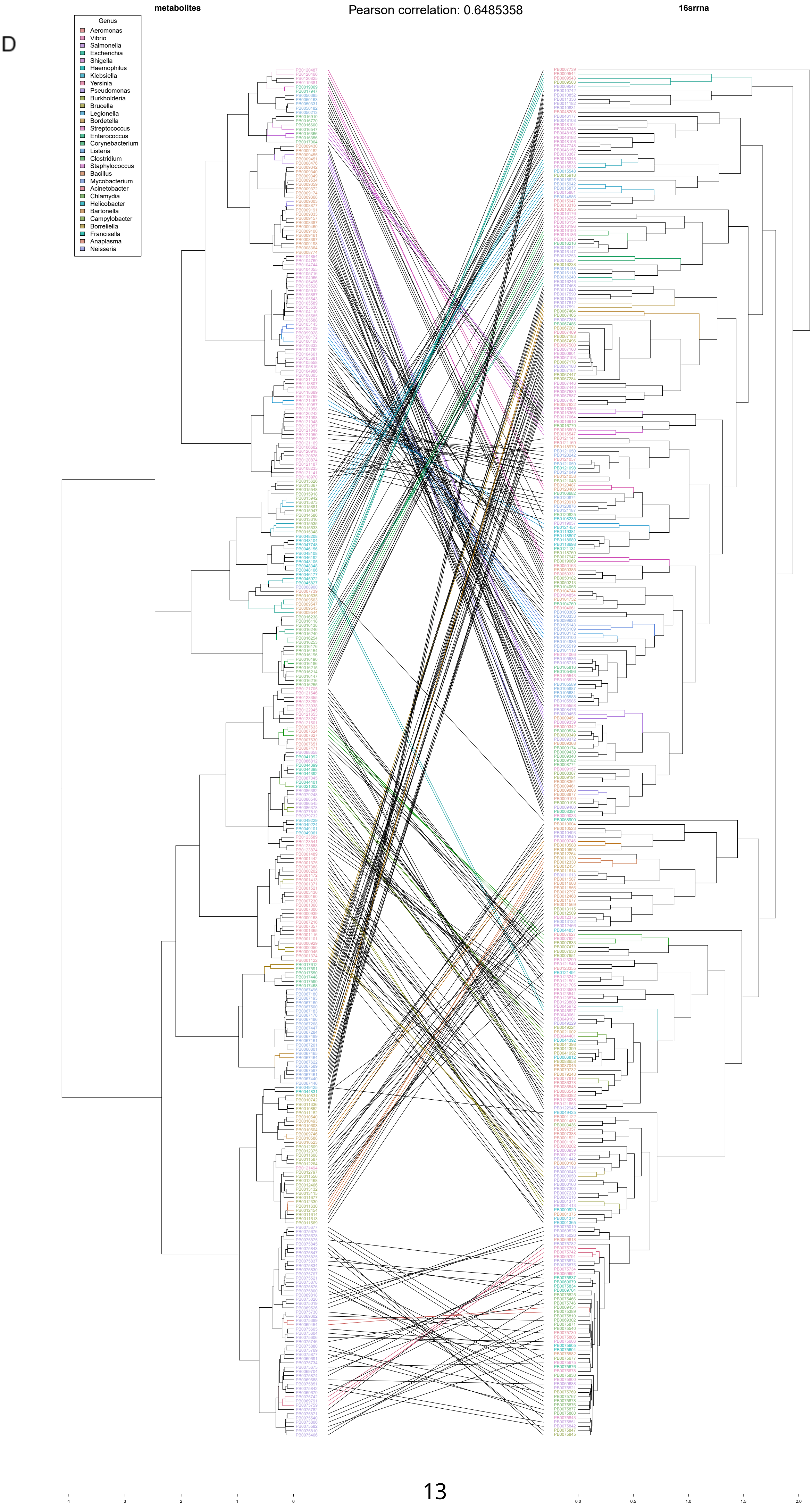
D
metabolites
Pearson correlation: 0.6485358
16srrna
Genus
Aeromonas
Vibrio
Salmonella
Escherichia
Shigella
Haemophilus
Klebsiella
Yersinia
Pseudomonas
Burkholderia
Brucella
Legionella
Bordetella
Streptococcus
Enterococcus
Corynebacterium
Listeria
Clostridium
Staphylococcus
Bacillus
Mycobacterium
Acinetobacter
Chlamydia
Helicobacter
Bartonella
Campylobacter
Borreliella
Francisella
Anaplasma
Neisseria
4 3 2 1 0
0.0 0.5 1.0 1.5 2.0

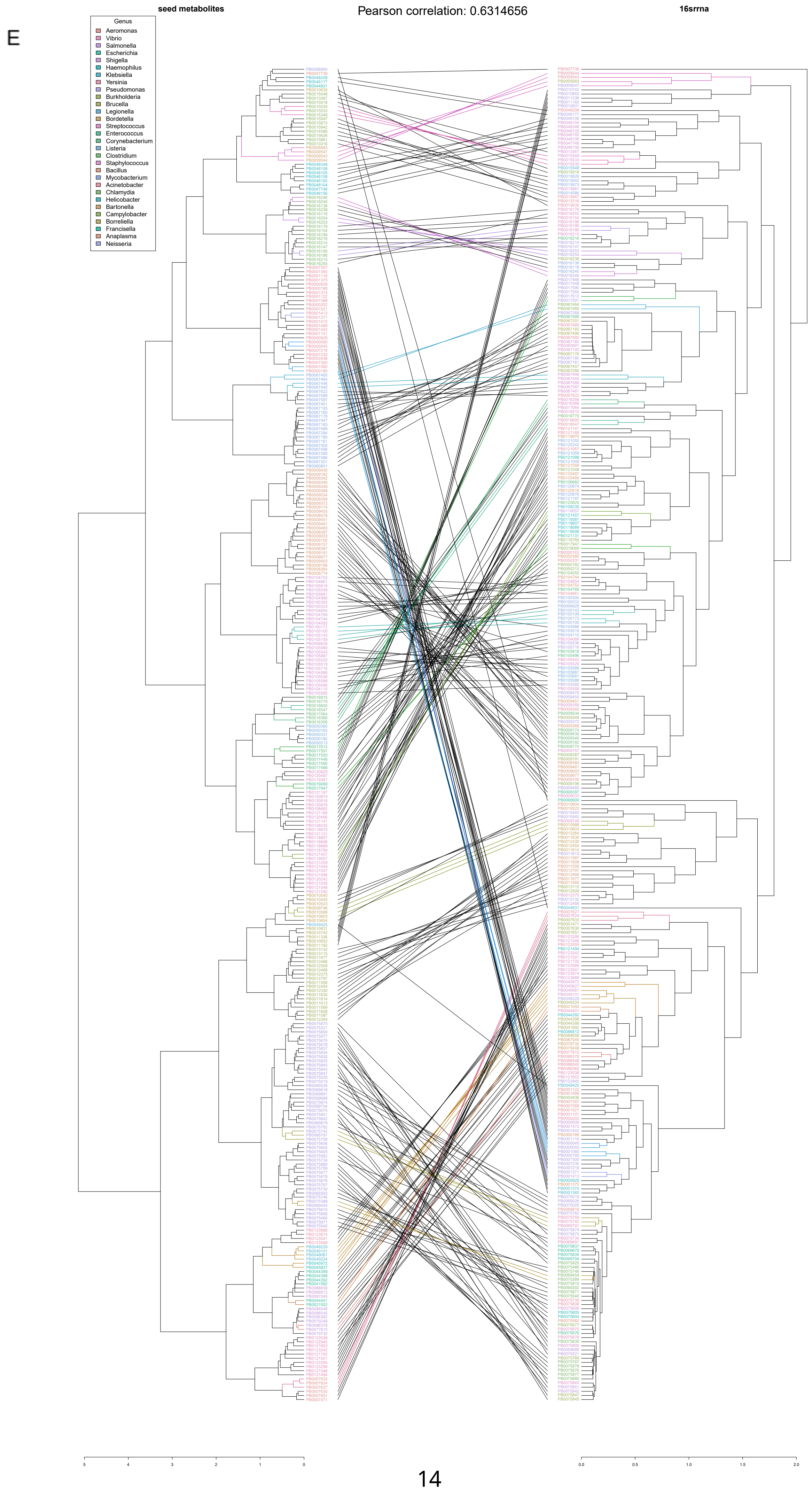
E
seed metabolites
Pearson correlation: 0.6314656
16srrna
Genus
Aeromonas
Vibrio
Salmonella
Escherichia
Shigella
Haemophilus
Klebsiella
Yersinia
Pseudomonas
Burkholderia
Brucella
Legionella
Bordetella
Streptococcus
Enterococcus
Corynebacterium
Listeria
Clostridium
Staphylococcus
Bacillus
Mycobacterium
Acinetobacter
Chlamydia
Helicobacter
Bartonella
Campylobacter
Borreliella
Francisella
Anaplasma
Neisseria
5
4
3
2
1
0
0.0
0.5
1.0
1.5
2.0

F

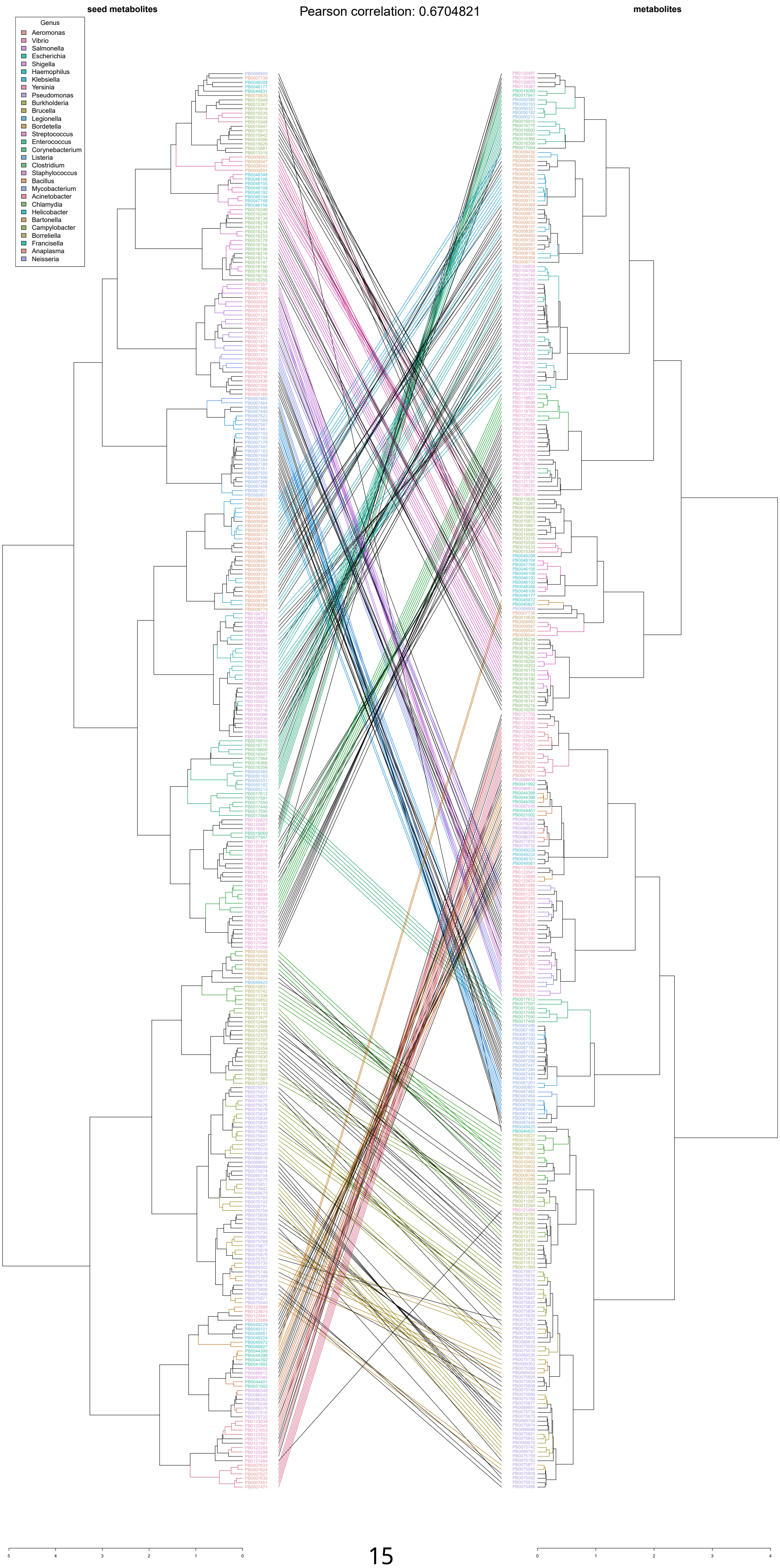
seed metabolites
Pearson correlation: 0.6704821
metabolites
Genus
Aeromonas
Vibrio
Salmonella
Escherichia
Shigella
Haemophilus
Klebsiella
Yersinia
Pseudomonas
Burkholderia
Brucella
Legionella
Bordetella
Streptococcus
Enterococcus
Corynebacterium
Listeria
Clostridium
Staphylococcus
Bacillus
Mycobacterium
Acinetobacter
Chlamydia
Helicobacter
Bartonella
Campylobacter
Borreliella
Francisella
Anaplasma
Neisseria
5
4
3
2
1
0
0
1
2
3
4

Fig. S1. Tanglegrams with all samples to compare between trees of the datasets (Bray–Curtis dissimilarity and Ward's hierarchical clustering method): A)bar120 vs. 16S rRNA; B) bar120 vs. metabolites; C) bar120 vs. seed metabolites; D) 16srrna vs. metabolites; E) 16srrna vs. seed metabolites; F) seed metabolites vs. metabolites;
